# The application of antimicrobial peptides as growth and health promoters for swine

**DOI:** 10.1186/s40104-015-0018-z

**Published:** 2015-05-07

**Authors:** Hao Xiao, Fangyuan Shao, Miaomiao Wu, Wenkai Ren, Xia Xiong, Bie Tan, Yulong Yin

**Affiliations:** Observation and Experiment Station of Animal Nutrition and Feed Science in South-Central China, Ministry of Agriculture, Hunan Provincial Engineering Research Center for Healthy Livestock and Poultry Production, Key Laboratory of Agro-ecological Processes in Subtropical Region, Institute of Subtropical Agriculture, Chinese Academy of Sciences, Changsha, Hunan 410125 China; University of the Chinese Academy of Sciences, Beijing, 10008 China; Department of Microbiology, Molecular Genetics, and Immunology, University of Kansas Medical Center, Kansas City, KS 66160 USA; Hubei Collaborative Innovation Center for Animal Nutrition and Feed Safety, Wuhan Polytechnic University, Wuhan, 430023 China

**Keywords:** Antimicrobial peptides, Antibiotics, Applications, Swine

## Abstract

With the widespread ban on the use of antibiotics in swine feed, alternative measures need to be sought to maintain swine health and performance. Antimicrobial peptides (AMPs) are part of the nonspecific defense system and are natural antibiotics produced by plants, insects, mammalians, and micro-organisms as well as by chemical synthesis. Due to their broad microbicidal activity against various fungi, bacteria and enveloped viruses, AMPs are a potential alternative to conventional antibiotics for use in swine production. This review focuses on the structure and mechanism of action of AMPs, as well as their effects on performance, immune function and intestinal health in pigs. The aim is to provide support for the application of AMPs as feed additives replacing antibiotics in swine nutrition.

## Background

Antibiotics have been used in the swine industry for more than 50 years to improve growth and prevent infectious diseases. However, the misuse of antibiotics has caused many problems including the emergence of bacteria resistant to antibiotics and the potential of producing drug residues in meat products [1]. As a result, a global trend has emerged towards restriction of the inclusion of antibiotics in swine diets as a routine means of growth promotion. In response, a considerable amount of research has been focused on the development of alternatives to antibiotics to maintain swine performance and health.

Antimicrobial peptides (AMPs) are one of the most widely researched alternatives to conventional antibiotics. AMPs are potent, broad spectrum antibiotics which have been demonstrated to kill gram-negative and gram-positive bacteria, mycobacteria, viruses, fungi and even transformed or cancerous cells while having no effect on the cells of treated animals [2]. In recent years, studies on AMPs and their applications have become one of the hot spots in the areas of agricultural science, biology, medicine, and physiology as well as having potential applications in medicine and the food industry.

Supplementation with various antimicrobial peptides has been reported to have positive effects on performance, nutrient digestibility, the intestinal microflora, intestinal morphology and immune function in pigs [3-5]. This article provides an overview of AMPs, their categories and structure, mechanism of action and their potential applications in swine production.

### Structure and categories of antimicrobial peptides

AMPs are oligopeptides with a variable composition of amino acids and amino acid number (typically 6 to 100 amino acids). Based on the different sources, AMPs are divided into mammalian AMPs (e.g. defensin), amphibian AMPs (e.g. magainins), insect AMPs (e.g. cecropin), plant AMPs (e.g. thionin), and microbial AMPs (e.g. gramicidin and nisin). Based on their biological activities, AMPs can also be divided into antiviral peptides (e.g. defensins, and NP-1), antibacterial peptides (e.g. nisin, and pyrrhocoricin), antifungal peptides, and antiparasitic peptides [6].

AMPs are small, positively charged, amphipathic molecules which possess both hydrophobic and hydrophilic regions. Based on their secondary structure, AMPs are characterized as one of four types including α-helical, β-sheet (due to the presence of 2 or more disulfide bonds), alpha-beta and non-alpha-beta structure [7]. Because they consist solely of amino acids, it is very easy to modify the structure of AMPs. Chemical synthesis or recombinant expression systems can be used to produce fully synthetic peptides [6].

### Mechanism of antimicrobial activity of antimicrobial peptides

**Figure 1:**
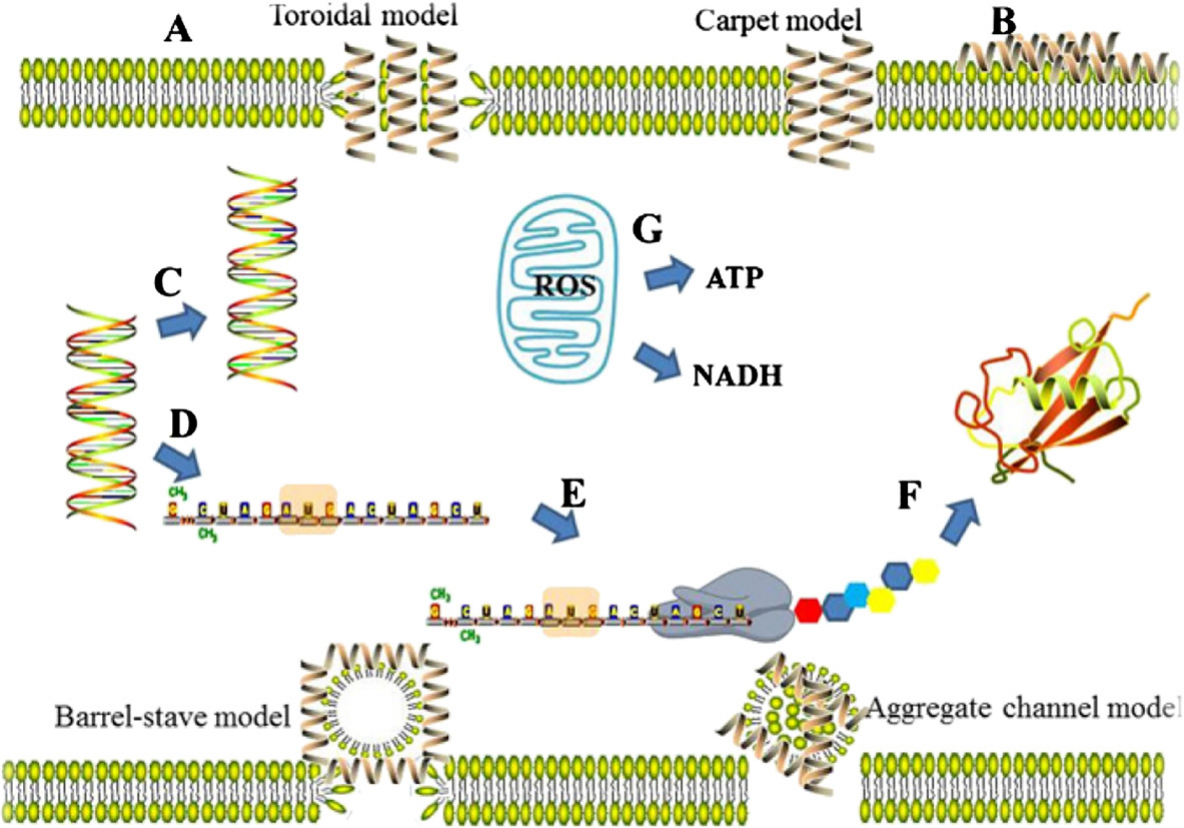
The diverse mechanistic modes of action for antimicrobial peptides. The figure means two main kinds of AMPs namely membrane-active AMPs and intracellular-active AMPs. The mechanism of membrane-active AMPs includes the “barrel-stave”, “toroidal”, “carpet” and “aggregate channel” models **(A)**; **B**-**G** means the mechanism of intracellular-active AMPs. **A**, membrane activity contains Toroidal model, Carpet model, Barrel-stave model and Aggregate channel model; **B**, Inhibition of enzymes necessary for linking of cell wall structural proteins; **C**, Inhibition of DNA synthesis; **D**, Inhibition of RNA synthesis; **E**, Inhibition of ribosomal function and protein synthesis; **F**, Blocking of chaperone proteins necessary for proper folding of proteins; **G**, inhibition of cellular respiration and induction of ROS formation and damage of mitochondrial cell membrane integrity and efflux of ATP and NADH.

It has been suggested that the interaction and action of AMPs with target cells depends on the two factors: the cell surface which is the classic and large acting mechanism and the amino acid composition of AMPs [8]. Furthermore, researchers have found that there have two main kinds of AMPs namely membrane-active AMPs and intracellular-active AMPs (Figure 1).

### Membrane-active antimicrobial peptides

In order to explain membrane disruption by AMPs, researchers have proposed many models including the “barrel-stave”, “toroidal”, “carpet” and “aggregate channel” models. Early in 1977, Ehrenstein and Lecar [9] proposed the “barrel-stave model” which suggests that peptides directly insert into the lipid core of the target membrane to form trans-membrane pores. In the “toroidal model”, peptide molecules are inserted into the membrane forming a bundle, inducing the lipid monolayers to continuously bend through the pore [9]. The “carpet model”, suggests that AMPs use a detergent-like action to cover the membrane surface in order to affect its architecture [10,11]. In the “aggregate channel model”, the peptides insert into the membrane and then cluster into unstructured aggregates that span the membrane. These aggregates are proposed to have water molecules associated with them providing channels for leakage of ions and possibly larger molecules through the membrane [12] (Figure 1).

### Intracellular-active antimicrobial peptides

Cell membrane permeabilization by AMPs was thought to be the primary mechanism of killing. However, there is increasing evidence to prove that some AMPs can interact with an array of intracellular targets including DNA, RNA and protein to kill their target cells, but not damage the cell membrane. AMPs can directly prevent DNA, RNA and protein synthesis, as well as cell wall synthesis and proteases of microbes by means of direct penetration and endocystosis to enter the cells [13] (Figure 1). For example, PR-39 from pig intestines can act like a proteolytic agent and suppress protein and DNA synthesis to kill bacteria [14], while seminaplasmin can inhibit RNA polymerase and stop RNA synthesis completely at very low concentrations [15].

### The applications of antimicrobial peptides in swine nutrition

Some AMPs, including antimicrobial peptide A3, P5, colicin E1, cecropin AD, and cipB-lactoferricin-lactoferrampin (cipB-LFC-LFA) have been shown to have beneficial effects on performance, nutrient digestibility, intestinal morphology as well as intestinal and fecal microflora. Their activities are summarized in Table 1.

**Table 1:** **Summary of studies showing the applications of AMPs in swine nutrition**

**Antimicrobial peptide and dose**	**Animal and treatments**	**Application effects**	**References**
Antimicrobial peptide-A3 (AMP-A3); 60 or 90 mg/kg	Weanling piglets fed with basal diet + 1.5 g apramycin/kg diet and basal diet supplemented with 0, 60 and 90 mg AMP-A3/kg diet in 2 phases (d 0–14 post-weaning: 14.28 MJ/kg ME and 15.5 g/kg lysine; d 15–28 post-weaning: 14.11 MJ/kg ME and 13.5 g/kg lysine)	Has beneficial effects on performance, total tract apparent digestibility of nutrients, intestinal morphology and intestinal and fecal microflora	Yoon et al. 2012 [19]
Antimicrobial peptide-P5 (AMP-P5); 40 or 60 mg/kg	Weanling piglets fed with basal diet, basal diet + 1.5 g/kg apramycin, basal diet + 40 mg/kg AMP-P5 and basal diet + 60 mg/kg AMP-P5 in 2 phases (d 0–14 post-weaning: 14.28 MJ/kg ME and 15.5 g/kg lysine; d 15–28 post-weaning: 14.11 MJ/kg ME and 13.5 g/kg lysine)	Improves the performance and apparent total tract digestibility of nutrients and reduces coliforms	Yoon et al. 2013 [4]
Synthetic antimicrobial peptide-A3 or P5 (AMP-A3 and P5); 60 mg AMP-A3 or 60 mg AMP-P5/kg	Weanling piglets fed with basal diet, basal diet + 150 mg/kg avilamycin, basal diet + 60 mg/kg AMP-A3 and basal diet + 60 mg/kg AMP-P5 for 28 days	Improves the performance, nutrient digestibility, intestinal morphology and to reduces pathogenic bacteria	Yoon et al. 2014 [20]
Antimicrobial peptide colicin E1; 11 or 16.5 mg/kg	Weaned pigs fed with diets containing 0, 11, or 16.5 mg colicin E1/kg diet and were orally inoculated with 1 x 10^9^ CFU of each of two F18-positive *E. coli* strains	Improves the performance, reduces incidence of postweaning diarrhe	Cutler et al. 2007 [21]
Antimicrobial peptide cecropin AD; 400 mg/kg	Weaned barrows fed with basal diet or similar diets supplemented with antibiotics (100 mg/kg kitasamycin plus 800 mg/kg colistin sulfate) or 400 mg/kg cecropin AD and were orally challenged with 10^9^ CFU/mL of *E. coli* K88	Enhances pig performance through increasing immune status and nitrogen and energy retention as well as reducing intestinal pathogens	Wu et al. 2012 [27]
cipB-lactoferricin-lactoferrampin (cipB-LFC-LFA); 100 mg/kg	Weanling piglets were challenged with enterotoxigenic *E. coli* and randomly assigned to four treatment groups fed a maize–soyabean meal diet containing either no addition, cipB at 100 mg/kg, cipB-LFC-LFA at 100 mg/kg or colistin sulfate at 100 mg/kg for 3 weeks	Improves performance through an antibacterial effect, the regulation of immune function, improvement of the absorption of Fe and a reduction in the incidence of diarrhea	Tang et al. 2009 [5]
Recombinant Lactoferrampin-Lactoferricin; 100 mg/kg	Weanling piglets fed with basal diet, basal diet + 0.1 g /kg lactoferrampin-lactoferricin and basal diet + 0.1 g /kg chlortetracycline for 21 days	Improves performance and affects serum parameters	Tang et al. 2012 [17]
Composite antimicrobial peptides (CAP, consist mainly of antibacterial lactoferrin peptides, along with plant defensins and active yeast); 400 mg/kg	Weanling piglets fed with basal diet, basal diet + 0.4% CAP, basal diet + 4 mg/kg deoxynivalenol, and basal diet + 4 ppm deoxynivalenol + 0.4% CAP for 30 days	Improves feed efficiency, immune function, and antioxidation capacity and alleviates organ damage	Xiao et al. 2013 [29]
Composite antimicrobial peptides (CAP, consist mainly of antibacterial lactoferrin peptides, along with plant defensins and active yeast); 400 mg/kg	Weanling piglets fed with basal diet, basal diet + 0.4% CAP, basal diet + 4 mg/kg deoxynivalenol, and basal diet + 4 ppm deoxynivalenol + 0.4% CAP for 30 days	Improves intestinal morphology and intestinal epithelial cell proliferation and protein synthesis; May repair the intestinal injury induced by DON	Xiao et al. 2013 [30]
Composite antimicrobial peptides (CAP, consist mainly of antibacterial lactoferrin peptides, along with plant defensins and active yeast); 400 mg/kg	Weanling piglets fed with basal diet, basal diet + 0.4% CAP, basal diet + 4 mg/kg deoxynivalenol, and basal diet + 4 ppm deoxynivalenol + 0.4% CAP for 30 days	Attenuate the metabolic disturbances in AA, lipid, and energy metabolism induced by DON.	Xiao et al. 2015 [31]
A mixture of lactoferrin, cecropin, defensin, and plectasin	Pigs fed with basal diet, basal diet + 2.0 g/kg of AMPs and basal diet +3.0 g/kg of AMPs for 32 days	Improves performance, reduces the incidence of diarrhea, and increases the survival rate of weaned pigs	Xiong et al. 2014 [32]

### Antimicrobial peptides can promote the performance of pigs

Antimicrobial lactoferrin peptides are one of the most prevalent AMPs used in swine nutrition. It has been demonstrated that dietary supplementation with recombinant lactoferrampin-lactoferricin (produced by the Institute of Subtropical Agriculture, Chinese Academy of Science which obtained through the expression of the lactoferampin-lactoferricin gene in the expression host P. *pastoris* (KM71) XS10 [16]) increased the final body weight and the average daily gain (ADG) of piglets by 13.3 and 29.3%, respectively while decreasing feed conversion by 11.5% [16,17]. Tang et al. [16,17] showed that piglets supplemented with cipB-LFC-LFA had higher ADG and ADFI than pigs fed control diets A mixture of AMPs including lactoferrin, cecropin, defensin, and plectasin was shown to enhance ADG, ADFI and G:F on 5 farms [18]. Growth promoting effects of the antimicrobial peptides A3 and P5 were also observed [4]. Increasing the levels of dietary AMP-A3 from 0 to 90 mg/kg in diets linearly improved ADG [19] while dietary supplementation with 60 mg/kg AMP-P5 increased ADG, ADFI and G:F [4], but the effects of AMP-A3 or P5 did not surpass that of a positive control treatment supplemented with 150 mg/kg avilamycin [20].

Dietary inclusion of Colicin E1 had a significant effect on pig performance in that pigs fed the control diet gained an average of 380 g, while pigs receiving 11 and 16.5 mg Colicin E1 per kg of diet gained 540 and 940 g, respectively [21]. However, the joint use of antibacterial peptide and Zn-Met did not show any synergistic effects on pig performance [22].

The effects of AMPs on performance can be explained on the basis of their antimicrobial activity. For example, Colicins E1 and N have been shown to inhibit the activities of *E. coli* strains that caused post-weaning diarrhea and edema disease in pigs [23]. The improvement in performance can also be related to improvements in nutrient digestibility [4,20,24]. Yoon et al. [4,19,20] found that pigs diets supplemented with AMP-A3 or P5 showed an increase in the apparent total tract digestibility of dry matter, crude protein and gross energy.

### Antimicrobial peptides can enhance the immune status of pigs

AMPs are important components of the host’s defense system and are effector molecules of innate immunity with direct antimicrobial and immune mediator function [2,25,26]. Tang et al. [5] found that dietary supplementation with cipB–lactoferricin–lactoferrampin increased serum IgA and IgG but reduced serum IgM. The researchers from National Feed Engineering Technology Research Center (Beijing, China) prepared antimicrobial peptide cecropin AD using cecropin A and cecropin D isolated from the silkworm Hyalophora cecropia and added it to weaned piglets challenged with *E. coli* [27]. The results show that cecropin AD could increase levels of secretory IgA in jejunum and serum IgA, IgG, interleukin-1β and interleukin-6 [27]. AMPs can influence the adaptive immune system, either directly or indirectly via alteration of the gut microflora [5]. This was confirmed by results showing that dietary AMP-A3 or P5 decreased fecal *Clostridium* spp. and coliforms, as well as decreasing ileal and cecal total anaerobic bacteria, *Clostridium* spp. and coliforms [20].

### Antimicrobial peptides can improve the intestinal health of pigs

A toxin produced by pathogenic bacteria in the gut can cause inflammation of the intestinal mucosa and diarrhea associated with morphological changes in the small intestine, such as shortening of the villi and an increase in crypt depth [28]. The antibacterial action of AMPs provides an effective support for normal intestinal morphology and function. Tang et al. [5] found that lactoferrampin-lactoferricin increased the height of the villi in the jejunum and ileum as well as the villus height: crypt depth ratio in the jejunum and ileum, which may be related to the fact that LFC-LFA can decrease the concentration of *E. coli* and increase lactobacilli and bifidobacteria in the gut. Similar results were observed in pigs following AMP-A3 [19] or cecropin AD [27] treatment. In addition, dietary supplementation with AMPs induced lower serum D-lactate concentrations [17] that increased intestinal permeability and enhanced the efficiency of absorption and utilization of nutrients.

### Antimicrobial peptides alleviate the toxic effects of deoxynivalenol (DON) in pigs

Recently, we found that AMPs played a protective effect in piglets challenged with DON [29]. The composite antimicrobial peptide GLAM®180# used in our studies contains antibacterial lactoferrin peptides, plant defensins and active yeast and these three bioactive components have been shown to have a positive effect on growth and health of animals. Feeding 0.4% GLAM®180# to piglets challenged with diets containing 4 mg/kg DON improved overall feed efficiency (Table 2), promoted blood circulation, alleviated organ damage, and reduced DON toxicity [29].

**Table 2:** **Effects of composite antimicrobial peptides on the performance of piglets (12–26 kg) challenged with** ***deoxynivalenol*** ^**1,2**^

**Item**	**Diets**				**SEM**	***P*** **-value**
	**NC**	**CAP**	**DON**	**DON + CAP**		
ADG, kg						
Day 0 to 15	0.32	0.24	0.30	0.30	0.01	0.09
Day 15 to 30	0.64^a^	0.66^a^	0.40^b^	0.48^b^	0.02	<0.01
Day 0 to 30	0.48^a^	0.45^a^	0.35^b^	0.39^b^	0.01	<0.01
ADFI, kg						
Day 0 to 15	0.67^a^	0.56^b^	0.68^a^	0.63^ab^	0.02	0.02
Day 15 to 30	1.33^a^	1.10^b^	1.07^b^	1.04^b^	0.03	<0.01
Day 0 to 30	1.00^a^	0.83^b^	0.87^b^	0.84^b^	0.02	<0.01
G:F						
Day 0 to 15	0.47	0.42	0.44	0.49	0.02	0.54
Day 15 to 30	0.49^b^	0.61^a^	0.38^c^	0.47^bc^	0.02	<0.01
Day 0 to 30	0.48^ab^	0.55^a^	0.40^b^	0.47^a^	0.01	<0.01

Xiao et al. 2013 [29].

^1^NC: Basal diet. CAP: Basal diet + 0.4% composite antimicrobial peptide, DON: Basal diet + 4 mg/kg DON, DON + CAP: Basal diet + 4 mg/kg DON + 0.4% composite antimicrobial peptide.

^2^ *n = 7.* ^a-c^ Values with different letters within the same row are significantly different (*P* < 0.05).

As indicators of intestinal morphology and function, the serum D-lactate and diamine oxidase content were lower but the villous height/crypt depth (Table 3) and the proliferating cell nuclear antigen (PCNA) labeling indexes in the jejunum and ileum were greater in piglets fed DON + GLAM®180# treatments than those in the DON treatment alone. In addition, GLAM®180# increased the protein levels of phosphorylated Akt, mTOR and 4E-binding protein 1 in the jejunum of piglets. The results indicate that GLAM®180# improved intestinal morphology and promoted intestinal epithelial cell proliferation and protein synthesis [30]. The combined results of ^1^H-NMR and LC-MS/MS showed the serum concentrations of HDL, unsaturated lipids, proline, citrate and fumarate were greater while those of glycoprotein, urea, TMAO, glycine and lactate were lower, in the DON + CAP group compared to those in the DON group, which indicated GLAM®180# could attenuate the metabolic disturbances in AA, lipid, and energy metabolism induced by DON [31]. The application of AMPs in DON challenged piglets demonstrates that GLAM®180# can alleviate the toxic effect of DON on pigs.

**Table 3:** **Effects of composite antimicrobial peptides on the jejunal and ileal morphology of piglets challenged with d** ***eoxynivalenol*** ^**1,2**^

**Item**	**Diets**				**SEM**	***P*** **-value**
	**NC**	**CAP**	**DON**	**DON + CAP**		
Jejunum						
Villus height, μm	240.01	239.08	197.56	221.87	7.05	0.23
Crypt depth, μm	126.39^ab^	117.03^b^	145.14^a^	110.48^b^	6.88	0.04
Villus height: Crypt depth	1.92	2.06	1.39	2.05	0.11	0.08
Goblet cell number	11.50	11.00	17.67	12.00	1.20	0.26
Lymphocyte number	195.00^b^	198.50^b^	256.33^a^	204.50^b^	9.26	<0.01
Ileum						
Villus height, μm	263.20^a^	240.15^a^	170.98^b^	185.08^b^	10.31	<0.01
Crypt depth, μm	117.73	120.05	109.45	104.73	4.36	0.56
Villus height: Crypt depth	2.32^a^	2.06^ab^	1.57^c^	1.81^ab^	0.09	0.04
Goblet cell number	22.33	16.00	19.00	18.50	1.36	0.38
Lymphocyte number	181.25^c^	154.75^d^	232.00^a^	204.00^b^	7.70	<0.01

Xiao et al. 2013 [30].

^1^NC: Basal diet. CAP: Basal diet + 0.4% composite antimicrobial peptide, DON: Basal diet + 4 mg/kg DON, DON + CAP: Basal diet + 4 mg/kg DON + 0.4% composite antimicrobial peptide.

^2^ *n = 7.* ^a-d^ Values with different letters within the same row are significantly different (*P* < 0.05).

## Conclusions

Due to their broad spectrum of activity against several species of bacteria, fungi, protozoa, and enveloped virus, AMPs show beneficial effects on performance, nutrient digestibility, intestinal morphology as well as intestinal and fecal microflora in pigs. With the development of technology, the cost of addition of AMPs is gradually reduced, especially in swine production. Although most AMPs did not provide equal effects to that of antibiotics in swine nutrition, they have considerable potential as an alternative for antibiotics in rations fed to swine.
